# Family Connectedness and Its Association With Sexual Risk-Taking Among Undergraduate Students at the University of Nairobi

**DOI:** 10.24248/EAHRJ-D-18-00027

**Published:** 2019-07-30

**Authors:** Florence WN Wachira, Muthoni Mathai, Dammas M Kathuku

**Affiliations:** a Department of Psychiatry, University of Nairobi, Nairobi, Kenya

## Abstract

**Background:**

Universities have a student population in the age range of 17 to 25 years, 75 % of whom are sexually active, with the median age of sexual debut at age 18 years. About half of all students are involved in risky sexual behaviour. Many interventions have decreased sexual risk behaviour in the short-term, but there is need for multilevel prevention, including targeting improvements in family relationships for sustained change. Perceived positive family connectedness has been found to be related to reduced sexual risk-taking among adolescents and young adults.

**Methods:**

This cross-sectional study evaluated the family connectedness and sexual behaviour of students aged 18 to 24 years at the University of Nairobi. There were 904 participants, both male and female, who were registered students of the University of Nairobi. After institutional and individual consent were granted, participants completed a self-administered questionnaire within their classes. The family subscale of the Hemingway Measure of Adolescent Connectedness was used to evaluate connectedness, and a sexual behaviour questionnaire was used to evaluate sexual risk-taking behaviour.

**Results:**

Six hundred forty (70.8%) of the respondents were sexually active – 372 males and 268 females. High-risk sex was reported by 203 male respondents (54.6%) and 117 females (43.7%). Reportedly abstinent participants had higher family connectedness scores than those who were sexually active (P<.001), and participants who reported less sexual risk-taking had higher mean family connectedness scores than those with higher sexual risk-taking (P<.001).

**Conclusion:**

Family connectedness had a significant influence on sexual risk-taking, and investment in family relationships could reduce risky sexual behaviour and potentially other risky behaviours among young adult university students.

## INTRODUCTION

There are about 5,000 new HIV infections per day, and 66% of these are in sub-Saharan Africa. About one-third of new HIV infections are among youth aged between 15 and 24 years, according to the 2018 Joint United Nations Programme on HIV/AIDS (UNAIDS) estimates. Of the new infections in Kenya, 93.7% are through sexual transmission.^1^ Over 20% of youth in Kenya initiate sexual activity before 15 years of age, and the median age of sexual debut is 18 years. Additionally, 18% of Kenyan females begin childbearing in their teens.^2^

Sexual risk-taking behaviour refers to having unprotected sex, having sex with multiple partners, and early onset of sexual activity before the age of 19 years. Sexual risk-taking among the youth is associated with unplanned pregnancies, abortions, school dropout, sexually transmitted infections, and long-term consequences to individuals and society.^3^

Among university students in Kenya, risky sexual behaviour was reported by about half of all participants of the HIV and AIDS Sero-Behavioural Study in 2010. This included multiple sexual partners, the influence of drugs and alcohol on unintended sex, and intergenerational sex. Among those testing positive for HIV, over 40% were influenced by drugs or alcohol to have undesired sex.^4^ A study conducted in Tanzania found that 31.8% of 600 individuals aged 15 to 24 years were involved in high-risk sexual behaviour.^5^ A Ugandan study investigating sexual decision making among adolescents found that influences included social pressure, cultural barriers to condom use, knowledge about HIV transmission and prevention, and compunctions about premarital sex.^6^ In Soweto, South Africa, about half (52%) of 16- to 18-year-old participants in a study investigating condom use were sexually active, and the average age at sexual debut was 16 years among males and 17 years among females. One-third of the sexually active adolescents reported inconsistent or absent condom use. Sexually active participants also tended to report earlier sexual debut; there was also a high prevalence of sexual partners older than 21 years of age.^7^

In Kenya, resources have been directed at various sexual risk behaviour-prevention strategies. These include sex education starting in primary school; voluntary counselling and testing (VCT) services for HIV in the community, health facilities and tertiary educational institutions; easy access to free condoms within tertiary institutions; a compulsory HIV/AIDS course in most universities; nation-wide campaigns promoting abstinence, condom use, and contraception; and multiple peer group endeavours.

However, it seems as though knowledge does not do much to change behaviour. For example, there was no significant difference between the 2008–09 Kenya Demographic and Health Survey (KDHS) and the 2014 KDHS in terms of the number of people with multiple sexual partners (2 or more) in the 12 months leading up to the survey, despite the aforementioned campaigns.

For sustained sexual risk behaviour change, multilevel interventions that go beyond knowledge and beliefs are necessary,^8^ including preventative interventions targeting improvements in family relationships^9^ and understanding cultural and family dynamics.^1^

Connectedness refers to the relationships that individuals have with others and the benefits of these relationships to the individual and society. Connectedness has been found to correlate with self-esteem, social interest, academic attitude, resilience, and protective factors, while disconnection has been found to be associated with substance use, violence, social skill deficits, and academic underachievement.^10^ Connectedness has also been found to be positively associated with overall mental health,^10^ future orientation,^11^ emotional resilience,^12^ and to be protective against depressed mood.^13^

Research conducted in the United States has shown that positive family connectedness is associated with reduced sexual risk-taking behaviours among high-risk adolescents^14^ and young adults.^15^ However, there is a paucity of published research on this topic, particularly from sub-Saharan Africa. This study, therefore, aimed to investigate the impact that family connectedness has on the sexual risk-taking behaviour of university students.

## METHODS

### Study Design

This was a cross-sectional descriptive study carried out at the University of Nairobi to establish the relationship between family connectedness and sexual risk-taking behaviour among students.

### Study Site

The university was selected by purposive sampling on the basis of being within Nairobi – for ease of data collection, having students from all over the country and from a variety of cultural and socioeconomic backgrounds, and having a wide range of academic programmes so as to provide a balanced sample population of young adults pursuing university education.

### Participants

Eligible participants were University of Nairobi students aged between 18 and 24 years who provided informed consent. Those who did not have at least 1 living sibling and at least 1 living parent or adult guardian were excluded.

### Sample Size

We determine the smallest sample size (n=841) required to allow for adequate statistical power using the following formula for estimating mean family connectedness score (family connectedness is a continuous outcome variable):

$$n=\frac{Z_{1-\propto }^{2}\sigma^{2}}{d^{2}}$$

Where, Z_1-α_ is 1.96 for the 95% confidence level; σ, 0.74, is the standard deviation of mean family connectedness score among young people^10^; and *d*, 0.05, is the margin of error for estimating the mean.

### Sampling Technique

We conducted multistage stratified random sampling to select the school or faculty and department. A school or faculty was randomly selected from each of the 6 colleges that make up the University of Nairobi, and a department was randomly selected from each of the randomly selected schools or faculties, where applicable. The number of students selected from each school or department was determined proportionately to achieve the required sample size; in other words, schools or departments with more students had a larger representation within the study sample. Classes within the selected schools and faculties were selected randomly, and all students within these classes were provided with questionnaires. Data were collected through questionnaires completed by randomly selected consenting students.

### Pilot Survey

The data collection instrument was piloted using a sample of 10 eligible students. The instrument had a reliability coefficient (Cronbach's alpha) of 0.91. The instrument was reviewed by 2 consultant psychiatrists from the University of Nairobi, and the content was found to be valid.

### Study Instrument

The anonymous questionnaire queried participant biodata and current living situation. There were 11 items about family (parent and sibling) connectedness drawn from the Hemingway Measure of Adolescent Connectedness (HMAC; items 4, 5, 14, 15, 24, 25, 34, 35, 36, 44, 45 in the HMAC), and 15 items about sexual behaviour.

Family connectedness items were rated on a 5-point Likert scale (not at all, not really, sort of, true, very true). Question 34 was reverse coded (1 was very true, and 5 was not at all). The lowest possible score was 1, and the highest possible score was 5 per question. For each section (parents connectedness, sibling connectedness), a mean score between 1 and 5 was calculated for comparison and analysis purposes.

Sexual risk indicators included early sexual debut; multiple sexual partners; unprotected sex; having sexual partners who are more than 5 years older; transactional sex; unintended sexual intercourse influenced by alcohol or substance use; being in a noncommitted, noncohabiting sexual relationship; and experiencing consequences of sexual risk-taking, including transmitted infections and unwanted pregnancies. These indicators were chosen from risk factors identified in other studies^3, 4^ and also from the Centers for Disease Control and Prevention (CDC) youth risk behaviour surveillance system. ^18^ For sexual risk-taking, the score per question was 0 to 5, where 0 meant no sexual risk (have never had sex before) and 5 was the highest risk; the lowest possible score was 0, and the highest possible score was 57.

The HMAC was developed as a psychometric measure of adolescent connectedness. It assesses present versus future orientation as well as connectedness to conventional worlds (parents, religion, school) and unconventional worlds (peers, neighbourhood, self).^10^ Among the 4 subscales of the HMAC, this study investigated the *Family (Parents and Siblings)* sub-scale. Validation studies demonstrated that the HMAC has satisfactory test–retest and inter-item reliability and convergent validity across samples.^16^ The HMAC subscales are invariant across gender and ethnicity and are, therefore, appropriate for most assessments.^17^

We generated the sexual behaviour questionnaire items with the aid of the co-authors, who are supervisors from the University of Nairobi colleagues. These items were adapted from the CDC youth risk behaviour surveillance system's sexual behaviour questions.^18^

### Data Management and Analysis

The data were collected using coded questionnaires and entered into a Microsoft Access (Microsoft Corp., Redmond, WA, USA) database. We analysed the data using IBM SPSS Statistics for Windows, Version 21.0 (IBM Corp. Armonk, NY, USA).

Descriptive characteristics were analysed and presented as percentages for categorical variables and means ± standard deviations or medians with interquartile ranges for continuous data.

Family connectedness was analysed following the guidance of the HMAC scoring manual. Mean family connectedness scores (± standard deviations) were calculated to compare between categories of participants.

Median sexual risk-taking behaviour scores were calculated for 2 categories of participants: (1) those with “low” risk-taking scores, that is, with a score below the median, and (2) those with “high” risk-taking scores above the median. The median was chosen as a central tendency measure to separate the high and low risk scores because sexual risk-taking is an ordinal variable, and the intervals in the order are not equal and cannot be quantified, making the median a robust comparator in the presence of outliers.^19,20^

Mean family connectedness scores were used to compare low-risk students with higher-risk students using Student's t-test. All the statistical tests were interpreted at the 5% level of significance. Linear multiple regression analysis was done with sexual risk-taking as the dependent variable and gender and family connectedness as independent variables.

## RESULTS

### Sociodemographic Characteristics

Of the 1,150 students who were screened, 904 fit the inclusion criteria; 52.8% were males, and 47.2% were females. The mean age of the participants was 21.3 ± 1.7 years. First-year students made up 28.3% of participants, 14.5% were in second year, 15.7% were in third year, and 35.6% were in fourth year. The majority of the students (71.9%) lived away from home, while 230 of the participants lived at the home of their parents or guardians.

### Family Connectedness

The mean parental connectedness score was 4.1 ± 0.6, and the mean sibling connectedness score was 4.0 ± 0.8.

There were significant differences in parental (*P*=.001) and sibling (*P*<.001) connectedness between the genders, with the females having higher mean connectedness scores. There was no significant difference in the mean parental and sibling connectedness scores based on residence (home versus away from home).

### Engagement in Sexual Activity

About two-thirds of the respondents, that is, 640 (70.8%) students reported having had sexual intercourse in the past, and 264 (29.2%) participants reported that they had never had sex. The mean age of the respondents who had never engaged in sexual intercourse, 20.8 years, was 0.8 years younger than those who had engaged in sexual intercourse (21.6 years; *P*<.001).

Males were 2.1 times more likely to have engaged in sexual intercourse than females (odds ratio [OR], 2.1; 95% confidence interval [CI], 1.6 to 2.8; *P*<.001). There was no significant difference in the residential status (home versus away from home) of participants who had ever engaged in sexual intercourse and those who had not. The likelihood of having engaged in sexual intercourse gradually increased with advancement in year of study, with 60.2% of first-year students having ever had sex and 80.1% of fourth-year students having ever had sex (OR, 2.7; 95% CI, 1.8 to 3.9; *P*<.001).

### Sexual Risk-Taking

The mean sexual risk-taking score was 26.1, and the median score was 25 (interquartile range, 21-30).

Males were more likely than females to engage in high-risk sexual behaviour (OR. 1.6; 95% CI, 1.1 to 2.1; *P*=.007). There was no significant difference in age, residential status, or year of study between participants who had high sexual risk-taking scores and those who had low sexual risk-taking scores.

### Family Connectedness and Engagement in Sexual Activity

Among participants who reported never having had sex, the mean parental connectedness score 4.3 ± 0.5, and the mean sibling connectedness score was 4.2 ± 0.7. Among those who had ever had sex, the mean parental connectedness score was 4.0 ± 0.7, and the mean sibling connectedness was 3.9 ± 0.8. Connectedness to both parents and siblings was significantly higher among participants who had never had sex compared to those who had reported having previously engaged in sexual intercourse (*P*<.001 for both).

### Family Connectedness and Sexual Risk-Taking

Among participants who had low sexual risk-taking scores, the mean parental connectedness score was 4.2 ± 0.5, and the mean sibling connectedness score was 4.1 ± 0.7. Among participants who had high sexual risk-taking scores, the mean parental connectedness score was 3.9 ± 0.8, and the mean sibling connectedness score was 3.7 ± 0.9. The mean parental and sibling connectedness scores were significantly lower for participants with high sexual risk-taking scores compared with those who had low sexual risk-taking scores (*P*<.001 for both).

### Family Connectedness and Gender as Predictors of Sexual Risk-Taking

In the multiple regression model, gender and connectedness were significant as independent factors of high sexual risk-taking. Male participants were 1.5 times more likely than females to be involved in high-risk sex (95% CI, 1.0 to 2.1; *P*=.035).

A unit increase in parental connectedness contributes 0.6 odds of sexual risk-taking (95% CI, 0.44 to 0.84); that is, it is associated with a 40% reduction in sexual risk-taking. A unit increase in sibling connectedness contributes 0.75 odds of sexual risk-taking (95% CI, 0.58 to 0.97), or a 25% reduction in sexual risk-taking.

## DISCUSSION

Most participants were sexually active, and high-risk sex was reported by just over half of the male participants and 44% of the females. Abstinent participants had higher family connectedness scores than sexually active participants, including those with lower sexual risk-taking scores.

The mean parental connectedness score was 4.1, and the mean sibling connectedness score was 4.0. These mean scores were slightly higher than the representative family connectedness statistics quoted for African American and Hispanic youth in the HMAC scoring manual, which are higher than the scores quoted for Caucasian American adolescents.^10^ The differences can be explained by the disparate cultural contexts of our study and the studies informing the HMAC. Females had higher mean parental and sibling connectedness scores than males, which was similar to the gender difference reported in the HMAC manual among Caucasian youth. The HMAC manual reports an inverse gender comparison, however, for African American and Latino American adolescents.^10^

The majority (n=640, 70.8%) of participants in our study were sexually active, with a larger proportion of the males having ever engaged in sex. Additionally, high-risk sex was reported by 54.6% of males and 43.7% of females. The likelihood of participants reporting past sexual intercourse gradually increased with advancement in year of study. These findings regarding the sexual behaviour of university students are similar to the findings of the HIV and AIDS Baseline Sero-Behavioural Study in Six Universities in Kenya in 2010,^4^ which also investigated the habits of university students between 18 and 24 years of age.

**TABLE:** Family Connectedness and Its Association With Sexual Activity and Sexual Risk-Taking; and Predictors of High Sexual Risk-Taking

	Mean Score (SD)	%	OR (95% CI)
**Family connectedness**			
**Parents connectedness**			
Male	4.039 (0.7)	-	-
Female	4.187 (0.6)	-	-
**Sibling connectedness**			
Male	3.881 (0.9)	-	-
Female	4.150 (0.8)	-	-
**Ever had sex (N=904)**			
Male	-	78.0	2.1 (1.6–2.8)
Female	-	62.8	1.0
**Family connectedness score and sexual activity**			
**Parents connectedness**			
Ever had sex	4.035 (0.7)	-	-
Never had sex	4.283 (0.5)	-	-
**Sibling connectedness**			
Ever had sex	3.920 (0.8)	-	-
Never had sex	4.227 (0.7)	-	-
**Engagement in high-risk sex**			
Male	-	54.6	1.6 (1.1–2.1)
Female	-	43.7	1.0
**Family connectedness score and sexual risk-taking**			
**Parents connectedness**			
High-risk	3.893 (0.8)	-	-
Low-risk	4.178 (0.5)	-	-
**Sibling connectedness**			
High-risk	3.736 (0.9)	-	-
Low-risk	4.101 (0.7)	-	-
**Predictors of high sexual risk-taking**			
**Gender (male)**	-	-	1.5 (1.0–2.1)
Parent connectedness	-	-	0.61 (0.44–0.84)
Sibling connectedness	-	-	0.75 (0.58–0.97)

Abbreviations: CI, confidence interval; OR, odds ratio; SD, standard deviation

The proportion (23.6%) of participants involved with multiple sexual partners was higher than the national averages reported in the 2014 KDHS (1% for women and 13% for men).^2^ Notably, the KDHS surveyed participants between the ages of 15 and 64 years, which was much broader than our age range of 18 to 24 years, and individuals in this narrower age range are more likely to be involved in noncommitted relationships.

The gender differences in risky sexual behaviour support Darwin's sexual selection theory, which predicts that males tend to engage in more risky behaviour than females overall, with this inclination arising as efforts to court the opposite sex and being reinforced and propagated by natural selection. Male adolescents in Catalonia have also been reported to engage in risky sexual behaviour (more sexual partners and less condom use) more frequently than females. The Catalonian investigators postulated that this finding was probably explainable by socially reinforced male attitudes towards sex and risk-taking in general, making it more attractive for males to engage in sexual intercourse earlier and take greater risks.^21^ It has been shown that even in everyday situations, males tend to take more risks and that this is particularly true among single males, especially in the presence of females.^22^

Connectedness is about relationships with others and the benefits accrued from these relationships. It is associated with resilience and protective factors.^10^ We found associations between connectedness to family and sexual behaviour patterns, with abstinent participants scoring higher in terms of parental and sibling connectedness than participants who reported having had sex. Among participants who reported previously having had sex, those with higher sexual risk-taking behaviour scores had a lower mean parent and sibling connectedness scores than those who reported less risk-taking.

Other studies have shown family connectedness to be protective against early sexual debut and involvement in high-risk sexual behaviour,^14^ that family relationships affect the incidence of unplanned adolescent pregnancies^23^ as well as condom use among young adults.^15^

We have seen that the family microsystem, consisting of parents and siblings, can influence behaviour. In this case, the association between family connectedness and sexual risk-taking supports the ecological systems theory. These findings also support attachment theory, which posits that supportive family relationships improve self-regulatory mechanisms and reduce people's tendency to use risky behaviour as a coping mechanism.

### Limitations

Purposive sampling was used to select the study site, and university students may not be representative of all young adults in the same age group, so the results cannot be generalised. We also must consider the potential for recall bias and under-reporting and over-reporting of sexual behaviour by some respondents, as the data collection tool was a self-administered questionnaire.

## RECOMMENDATIONS

There is need to create a focus on improved family relationships as a key preventive measure against risky behaviour, particularly risky sexual behaviour. This should be incorporated into the discourse of policy-making and public education as well as premarital, marital, and parent counselling.

Family-centred interventions should also be created and implemented in the fight against the HIV/AIDS pandemic.

We found that females had higher family connectedness levels and abstinence rates, as well as lower levels of sexual risk-taking. There is need for further research to evaluate why females are more connected to their families and also on differences in male and female socialisation behaviours.

Owing to various constraints, this study was conducted at only 1 university in Nairobi. More research could be done involving youth from a wider array of institutions and settings, for a better understanding of risk-taking behaviour and its contributing factors among young adults.

Other sociocultural factors that may affect sexual risk-taking can also be studied; for example, the effects of poverty, substance abuse, and peer influences.

Further studies on connectedness, in its various facets, can be carried out to generate local and regional data, to investigate the relationship between connectedness and resilience, and to determine its effects on various aspects associated with risk-taking behaviour, including substance use, academic performance, and professional achievement.

## CONCLUSION

Most university students who participated in this study were sexually active, and a significant proportion of them reported engaging in high-risk sexual behaviour, potentially exposing them to sexually transmitted illnesses and unplanned pregnancies. The study also demonstrated that family connectedness might act as a protective factor against sexual risk-taking. Investing in family relationships is a viable avenue that needs to be explored as a mitigating factor for risky sexual behaviour.
